# Modern Sociology

**Published:** 1902-09-27

**Authors:** 


### Modern Sociology.

### POVERTY, HUNGER, AND DIRT.

The discussion at the British Medical Association on the relation between poverty and disease was interesting chiefly as showing the point of view from which different men regard the same problem. Neither Alderman McDougall, Mr. Rowntree, nor Dr. Drysdale denied the connection between poverty, disease, and death; at least none of them denied that the people who live in the poorer districts of Manchester, York, and London die in larger proportions than those in the richer; but Mr. Rowntree was well advised in dividing poverty into "primary" and "secondary" poverty. The first consists in actual lack of the necessaries of life, the second in that waste and thriftlessness which causes people who have a really sufficient income to lack necessaries because they spend money in things which are not necessary and perhaps even harmful. But it cannot be said that this division enables us to put the flattering unction to our souls that there is no real "primary" poverty, but only that which arises from waste, folly, and intemperance. Mr. Rowntree had gone carefully into the minimum amount of food required to keep a family consisting of a man, his wife, and three children in a state of full efficiency. The expenditure on rent he took at the lowest possible, for rent is one of the things in which the poor most generally economise, and his estimate for clothing, fire, and household repairs and sundries was also put at a very low figure. Thus taking every item of expenditure at a minimum, he reckoned that to maintain the family included in his estimate there was needed an income of not less than 21s. 8d. per week. "In order to ascertain how many families in York were living in a state of 'primary' poverty," said Mr. Rowntree, "the income of each was compared with the estimate, due allowance being made in every case for the size of family and rent paid." The result of this inquiry was to show that almost exactly 10 per cent. of the population of the city was living in a condition of "primary," that is, inevitable poverty.

It might be thought that this inevitable poverty would occur chiefly in the case of widows, or those in whose families the chief wage-earner was ill or too old to work. The death of the head of the family accounted for about 16 per cent. of the poverty, and his illness or old age to 5 per cent. more. Lack of work accounted for 2 per cent., irregularity of work for 3 per cent., while in 22 per cent. Mr. Rowntree reckoned that the cause of the poverty was that the family numbered more than four children—more than the income could comfortably keep. But in 52 per cent. of the cases the family was not larger than the estimated one—husband, wife, and three children—and the poverty was due to the fact that, although the wage-earner was in regular employment, his wages were insufficient to maintain his family in that degree of comfort which is essential to economic efficiency. "Adopting Atwater's standard for men doing moderate work as the standard, it was found that while the servant-keeping class consumed 15 per cent. more than the standard, and the artisan class earning over 26s. were adequately fed, the labouring-class families were seriously underfed. . . . They received 27 per cent. less food than is given to able-bodied paupers in workhouses, and 30 per cent. less than is given to convicts in English prisons. They also receive considerably less food than poor families in Philadelphia, Chicago and New York." The effect of this underfeeding on the efficiency of work needs little demonstration. "The curse of the poor is their poverty." Because a man does not earn enough to keep him in health and strength, his work will become less efficient as he grows older, and he will less and less be able to earn more. His average efficiency and his average wage will fall together. And what affects the health of the parent will affect both life and health in the child. More children will die in infancy and before they reach the wage-earning age, which—we put aside all considerations of family affection and human feeling, and take the matter solely from the economic standard—is a waste of the national resources. Every child fed and clothed for a certain number of years, who yet dies before repaying to his parents directly, from his wages, and to his country indirectly by his work, for the cost of his maintenance is so much loss—a bad national investment—perhaps the worst. And under these conditions live about 10 per cent. of the total population—for though Mr. Rowntree's statistics were taken from York, we cannot flatter ourselves that the labouring class is worse off in York than elsewhere.

"Secondary" poverty, though it does not command the same sympathy from the world, is equally bad in its effects on the children. And it is reckoned by Mr. Charles Booth that about 30 per cent. of the total urban population of England is living in poverty. The children, then, of nearly a third of our total urban population—and nowadays 77 per cent. of the total population dwells in towns—are brought up under conditions which tend to make them physically unfit for the struggle for life. Mr. Rowntree compared the children attending better-class schools with those who went to schools attended only by the poor, and found that the average height of the boys of the poor section when they leave school is 3½ inches less than that of their more favoured fellows, while the difference in weight in favour of the well-to-do is 11 lb. It is practically certain that the bulk of these must remain members of the poor and comparatively useless class in which they are born. We say useless in no unkindly spirit, but it is admitted that the class of the people which is poorest in quality both physically and mentally, get tasks assigned to them which their superiors in mind and body would scorn, and which, were there no degenerates at hand to do them, would probably be performed by machinery. Not man's necessity, but man's superiority and consequent scorn of drudgery, is invention's opportunity.

But the most imminent question for those who love their fellow men is not how to find mechanical substitutes for these poor creatures, but how to raise them so that in their own interest and that of their country they may be able to do work which no machine can do. Dr. Drysdale, Consulting Physician to the Metropolitan Hospital of London, who contributed the third paper to the discussion of the British Medical Association, advocated the limitation of families. But not only is this a remedy (if remedy it be) which does not appeal to British notions, but one which ignores the fact that in all classes of the community the birth-rate is decreasing rapidly. Prudence may be the reason of this in the middle classes, but among the poor, prudence is rather conspicuous by its absence, and if the birth-rate among the poor is falling, it is a result of this very physical weakness which is to be combated. Perhaps thereby the disease will cure itself, as in by-gone days the "Black Death" which killed so many of the labouring population raised the status of the survivors. The weakest will go to the wall, and the strongest will live and leave children behind them, according to nature's inflexible law. But in so far as man can meddle in the matter, it would seem that all we can do is to try still further to cheapen food and clothing, let charity help those children whose parents are unable to give them a sufficiency of comfort to provide for their development, and still further encourage those scientific inventions which increase the productiveness of labour.

